# Supplementary material for: Compact eternal diffractive neural network chip for extreme environments
Source: Commun Eng. 2024 May 1;3:64. doi: 10.1038/s44172-024-00211-6 (PMC11063033; doi:10.1038/s44172-024-00211-6)
Supplement: Supplementary file 2 — Supplementary Information [file 44172_2024_211_MOESM2_ESM.pdf]

# Supplementary Information

## Compact eternal diffractive neural network chip for extreme environments

Yibo Dong[1,3], Dajun Lin[1,2,3], Long Chen[1,2], Baoli Li[1], Xi Chen[1], Qiming Zhang[1], Haitao Luan[1*], Xinyuan Fang[1*], and Min Gu[1*].

[1]Institute of Photonic Chips, University of Shanghai for Science and Technology, Shanghai, 200093 China

[2]Centre for Artificial-Intelligence Nanophotonics, School of Optical-Electrical and Computer Engineering, University of Shanghai for Science and Technology, Shanghai, 200093 China

[3]These authors contributed equally.

Emails: haitaoluan@usst.edu.cn; xinyuan.fang@usst.edu.cn; gumin@usst.edu.cn

## Supplementary Text

### Note 1: Angular spectrum diffraction used in DNN propagation model

The input complex-amplitude field from the $n$-th neuron located at $(x_n, y_n)$ in the $l$-th layer to the $n$-th neuron in the ($l$+1)-th layer can be expressed as:

$$u_n^{l+1}(x, y) = F^{-1}\{F\{u_n^l(x_n, y_n)t_n^l(x_n, y_n)\}H(f_x, f_y)\} \tag{1}$$

where $F$ and $F^{-1}$ denote Fourier transform and inverse Fourier transform, respectively. The transmittance coefficient $t$ represents the amplitude and/or phase modulation of a neuron and can be expressed as $t_n^l(x_n, y_n) = A_n^l(x_n, y_n)\exp(\mathrm{j}\varphi_n^l(x_n, y_n))$, where $\mathrm{j} = \sqrt{-1}$. Quartz has a high transmittance in the visible light band, therefore, in our experiment, the DNN was trained to be phase type and $A$ was supposed to be a constant. $H(f_x, f_y)$ is the transfer function in spatial frequency domain and represents the phase delay after propagating a distance of $d$.

$$H(f_x, f_y) = \exp((j2\pi d/\lambda)\sqrt{1 - (\lambda f_x)^2 - (\lambda f_y)^2}) \tag{2}$$

where $f_x$ and $f_y$ are the spatial frequencies in the x and y direction, respectively and $\lambda$ is the wavelength of incident light. Therefore, by superimposing the complex-amplitude field propagated from all the neurons in the $l$-th layer, the total complex-amplitude field on the ($l$+1)-th layer can be obtained.

**Note 2: Training of the binary DNN**

The training of DNN is based on the Tensorflow 2.0 framwork. The input optical image is obtained by converting the grayscale value of the handwritten digit image to normalized amplitude value. The phase distribution in each layer are set to trainable tensors $\varphi$. The trained discrete phase distribution lowers the fabrication complexity. Directly discrete variables cannot be differentiated to generate the gradient during optimization. Here, we use a discrete phase training method inspired by a binary neural network (BNN)[1]. Similar to the trained weights and activations in the BNN, the variables (phase distribution) in our optimization can be also trained as 2-level or multi-level value. In forward model, the phase variables are deterministic:

$$\varphi^m = round(\varphi)/m \tag{3}$$

where $\varphi^m$ is the discrete phase, $m$ is the number of levels and $round()$ function in python returns a floating-point number rounded to the specified number of decimals. We additionally divide $m$ to constrain the variable value to 0 and 1. In backward model, the gradient of the discrete tensor can be defined by straight through estimator, described as:

$$g = \varphi + tf.stop\_gradient(\varphi^m - \varphi) \tag{4}$$

where $tf.stop\_gradient()$ is to restrict the flow of gradients through certain parts of the network. The returned value $g$ is constrained to be between 0 and 1 and can be scaled by the desired phases. In our experiment, the binary phases were chosen to be 0 and $\pi/2$.

We defined a mean squared error (MSE) as the loss function to evaluate the difference between the output of DNN and the desired target. The MSE was defined as follow

$$\text{MSE}=\frac{1}{n}\sum_{i=1}^{n}(I - I_i)^2 \tag{5}$$

where $I$ is the output of DNN and $I_i$ is the target intensity distribution at the output layer, $n$ refers to the number of measurement points at the output layer. We defined 10 regions on the output layer corresponding to the 10 types of handwritten digits. In our case, $n$=10 corresponds to the number of handwritten digit types.

In our experiment, 50 epochs were carried out in the training under the Tensorflow

framwork. 1000 images were used as the training set. A large number of neurons (1024×1024) were used, therefore, the spatial bandwidth product can be high to improve the output image quality. The distance between the input layer and the 1st diffractive layer was set to 5 cm. The distance between the 2nd diffractive layer and the output layer was set to 16.4 cm. Because the quartz plate we used is a commercial 2-inch single-crystal wafer with a thickness of 500 μm, the layer distance between the 1st and 2nd diffractive layer has to be fixed at 500 μm (about 940$\lambda$).

**Note 3: Analysis of the effect of the layer number on the performance of DNN**

We analyzed the effect of the layer number on the performance of DNN from the perspective of degree of freedom of the weight values. The degree of freedom is defined as the value constraints between the elements in the weight matrix. If there is no constraint between the elements, the degree of freedom is the highest.

First, we should note that the fundamental role of increasing the number of diffractive layers in DNN is different from that in deep neural networks of computer programs. In the deep neural networks, the output of each layer is processed nonlinearly by a non-linear activation function, so that each hidden layer can play a role. If there is no activation function, then all hidden layers can be combined into one linear layer, and increasing the number of layers will not make sense. For instance, in two hidden layers, the operation performed by each layer is as follows

$$y_1 = w_1 x_1 + b_1 \tag{5}$$

$$y_2 = w_2 y_1 + b_2 \tag{6}$$

where $x$ is the input matrix of each layer, $y$ is the output matrix and $b$ is the offset matrix. Then, the two equations can be combined and rewritten as follows.

$$y_2 = (w_2 w_1) x_1 + (w_2 b_1 + b_2) \tag{7}$$

We can see that a new hidden layer with $w_3 = w_2 w_1$ and $b_3 = w_2 b_1 + b_2$ can be used to replace the above two layers. Therefore, in the absence of an activation function, no matter how many hidden layers are used, the effect is the same as one layer.

For DNN, there is no nonlinear operation between diffractive layers. It seems that the use of multilayer in DNN is meaningless. However, in fact, increasing the number of layers of DNN still works. First, we can describe the complex-amplitude field propagating from a neuron in the $l$-th layer to another neuron in the ($l$+1)-th layer with the following Equation.

$$y^{l+1} = w^l t^l x^l \tag{8}$$

where $t$ is the transmittance coefficient same with that in Equation (1) and $x$ is the complex-amplitude field input into the neuron in the $l$-th layer. The $w$ is the complex-amplitude change produced during the propagating and can be obtained by Rayleigh-Sommerfeld diffraction[2]. According to Huygens' principle, we can get the propagation of the total complex-amplitude field in DNN.

$$\begin{bmatrix} x_1^{l+1} \\ x_2^{l+1} \\ \vdots \\ x_m^{l+1} \end{bmatrix} = \begin{bmatrix} w_{1,1}^l & w_{2,1}^l & \cdots & w_{n,1}^l \\ w_{1,2}^l & w_{2,2}^l & \cdots & w_{n,2}^l \\ \vdots & \vdots & \vdots & \vdots \\ w_{1,m}^l & w_{2,m}^l & \cdots & w_{n,m}^l \end{bmatrix} \left( \begin{bmatrix} t_1^l \\ t_2^l \\ \vdots \\ t_n^l \end{bmatrix} * \begin{bmatrix} x_1^l \\ x_2^l \\ \vdots \\ x_n^l \end{bmatrix} \right) \tag{9}$$

where $x_n^l$ refers the complex-amplitude field input of the $n$-th neuron of layer $l$, $t_n^l$ refers to the corresponding transmittance coefficient and $w_{n,m}^l$ refers to the diffraction from the $n$-th neuron in layer $l$ to the $m$-th neuron in layer $l$+1. The symbol "*" denotes the Hadamard product. Then, we can rewrite the Equation (9) as follows.

$$\begin{bmatrix} x_1^{l+1} \\ x_2^{l+1} \\ \vdots \\ x_m^{l+1} \end{bmatrix} = \begin{bmatrix} w_{1,1}^l t_1^l & w_{2,1}^l t_2^l & \cdots & w_{n,1}^l t_n^l \\ w_{1,2}^l t_1^l & w_{2,2}^l t_2^l & \cdots & w_{n,2}^l t_n^l \\ \vdots & \vdots & \vdots & \vdots \\ w_{1,m}^l t_1^l & w_{2,m}^l t_2^l & \cdots & w_{n,m}^l t_n^l \end{bmatrix} * \begin{bmatrix} x_1^l \\ x_2^l \\ \vdots \\ x_n^l \end{bmatrix} \tag{10}$$

From the above equation, the weight of DNN can be regarded as $w^l t^l$. The weight matrix $W_1$ can be written as follows.

$$W_1 = \begin{bmatrix} w_{1,1}^l t_1^l & w_{2,1}^l t_2^l & \cdots & w_{n,1}^l t_n^l \\ w_{1,2}^l t_1^l & w_{2,2}^l t_2^l & \cdots & w_{n,2}^l t_n^l \\ \vdots & \vdots & \vdots & \vdots \\ w_{1,m}^l t_1^l & w_{2,m}^l t_2^l & \cdots & w_{n,m}^l t_n^l \end{bmatrix} \tag{11}$$

Before training the DNN, we need pre-set parameters including the wavelength, layer distance, neuron size and number, so $w^l$ is a constant and $t^l$ is the only parameter we can train to optimize. We can see that the value of $t^l$ in each column is the same, therefore, once the value of one element in a column is determined, the other elements in this column will also be determined, which means that there is a strong correlation between the values of each column in the weight matrix. Relatively, in deep neural networks, the value of each element has no such relationship and can be trained to any value. In DNN training, the gradient descent algorithm is used to find the weight

value that minimizes the loss. The freer the value range of the weight is, the closer the loss after training is to the minimum value. Therefore, due to the existence of this unwanted constraint, the loss of monolayer DNN is bound to be higher than that of monolayer deep neural networks under the same number of neurons.

When we increase the DNN to bilayer, since there is no nonlinear activation function, the new weight matrix $W_2$ can be written as follows.

$$W_2 = \begin{bmatrix} w_{1,1}^{l+1}t_1^{l+1} & w_{2,1}^{l+1}t_2^{l+1} & \cdots & w_{n,1}^{l+1}t_n^{l+1} \\ w_{1,2}^{l+1}t_1^{l+1} & w_{2,2}^{l+1}t_2^{l+1} & \cdots & w_{n,2}^{l+1}t_n^{l+1} \\ \vdots & \vdots & \vdots & \vdots \\ w_{1,m}^{l+1}t_1^{l+1} & w_{2,m}^{l+1}t_2^{l+1} & \cdots & w_{n,m}^{l+1}t_n^{l+1} \end{bmatrix} \begin{bmatrix} w_{1,1}^{l}t_1^{l} & w_{2,1}^{l}t_2^{l} & \cdots & w_{n,1}^{l}t_n^{l} \\ w_{1,2}^{l}t_1^{l} & w_{2,2}^{l}t_2^{l} & \cdots & w_{n,2}^{l}t_n^{l} \\ \vdots & \vdots & \vdots & \vdots \\ w_{1,m}^{l}t_1^{l} & w_{2,m}^{l}t_2^{l} & \cdots & w_{n,m}^{l}t_n^{l} \end{bmatrix} \quad (12)$$

After multiplying the two matrices, we can obtain the first element of the weight matrix is as follows.

$$W_2(1,1) = w_{1,1}^{l+1}w_{1,1}^{l}t_1^{l+1}t_1^{l} + w_{2,1}^{l+1}w_{1,2}^{l}t_2^{l+1}t_1^{l} + \ldots + w_{n,1}^{l+1}w_{1,m}^{l}t_n^{l+1}t_1^{l} \quad (13)$$

Compare with the $W_1(1,1) = w_{1,1}^{l}t_1^{l}$ in Equation (11) of monolayer DNN, we can see that after changing DNN to bilayer, a new set of trainable parameters ($t_1^{l+1}$ to $t_n^{l+1}$) is added to each element in the weight matrix. Then, the values of the elements in each column in $W_2$ will no longer be fully constrained, so the loss of bilayer DNN can be optimized to be lower than that of monolayer.

According to Equations (13), we can give a quantitative definition to the degree of freedom of the weight value, that is, the number of trainable parameters $t$ in a weight matrix element. As the $t$ increases, the degree of freedom of the weight value increases. Therefore, we can conclude that the increase in the layer number of DNN helps to improve the recognition accuracy. However, at the same time, the increase in the number of layers can only reduce the value constraint and cannot eliminate this constraint. As a conclusion, no matter how many layers of DNN we used the recognition accuracy cannot exceed the that of monolayer deep neural networks under the same number of neurons. Increasing the number of layers of DNN can only make the recognition accuracy approach the accuracy of monolayer deep neural networks.

One important thing should be noted that since different training methods will also affect the accuracy of a neural network, the premise of this conclusion is under the same training method.

To verify this conclusion, we simulated the accuracy of DNN with different numbers of layers and monolayer deep neural networks (Supplementary Figure 5). The simulation results are in good agreement with this conclusion. Based on this conclusion, we can easily estimate the accuracy limit of a DNN with a certain number of neurons.

**Note 4: Analysis of the effect of layer distance on the performance of DNN**

The layer distance is also an important parameter that affects the performance of DNN. We simulated the accuracy of DNN under different layer distance (Supplementary Figure 6d). It can be seen that the accuracy of DNN will gradually increase as the layer distance increases.

We can also explain this phenomenon from the perspective of the degree of freedom of the weight value. The area of the zero-order diffraction will increase with the increase of the layer distance. Since the light intensity is mainly concentrated in the zero-order diffraction, the increase of the layer distance means that the number of neuron connections will increase. From Equation (13), it can be seen that there are $n$+1 trainable parameters that determine $W_2(1,1)$ under full connection, including $t_1^{l+1}$ to $t_n^{l+1}$ and $t_1^l$. There are also 2$n$ constants in $W_2(1,1)$, including $w_{1,1}^{l+1}$ to $w_{n,1}^{l+1}$ and $w_{1,1}^l$ to $w_{1,m}^l$. Among them, $w_{1,1}^l$ to $w_{1,m}^l$ respectively represent the complex-amplitude changes of the light from all the neurons in the 1st layer to one neuron in the 2nd layer.

When the layer distance is small, it means that some neurons in the two layers cannot build connections through zero-order diffraction. In our case, one neuron in the 2nd layer is mainly connected to 7×7=49 neurons in the 1st layer, so except for the $w$ corresponding to these 49 neurons, the absolute value of $w$ corresponding other neurons will be very small, so that the corresponding monomial in $W_2(1,1)$ can be ignored. This leads to a decrease in the actual number of trainable parameters for $W_2(1,1)$. Therefore, the degree of freedom of the weight value will decrease, resulting in the increase of the loss.

**Note 5: Analysis the impact that other extreme environments may have on the chip**

In addition to high temperatures, extreme environments for chips also include high-pressure environments, strong radiation environments, and high-humidity environments.

First, we analyze the high-pressure environment. The DNN chip was fabricated by surface carving on a single-crystal quartz wafer using plasma dry etching. This process does not alter the lattice structure of quartz, so we think that the chip's robustness under high pressure can be compared to that of a silica crystal without structures. Typically, the threshold for high pressure to alter the lattice of a silica crystal is above 25 GPa[3]. Changes in the crystal lattice will lead to changes in the refractive index, which can be regarded as the failure of the chip. Therefore, it is speculated that this chip can withstand high pressures above GPa. Additionally, in strong radiation environments, defects may form in quartz[4], which could scatter light. This scenario is similar to the experimental results obtained after high-temperature annealing (refer to Figure 6b and c in the Main Document). After annealing, the surface of the chip becomes rough, which scatters incident light as well. However, the inference accuracy of the chip is not affected. This is because the inference results are obtained by comparing the relative strength of the ten light spots. Therefore, we think that the radiation-induced defects in quartz will also not affect the chip's performance under certain radiation limits. Third, for high humidity environments, due to the high stability of silica, we think that the humidity will not affect the chip’s performance.

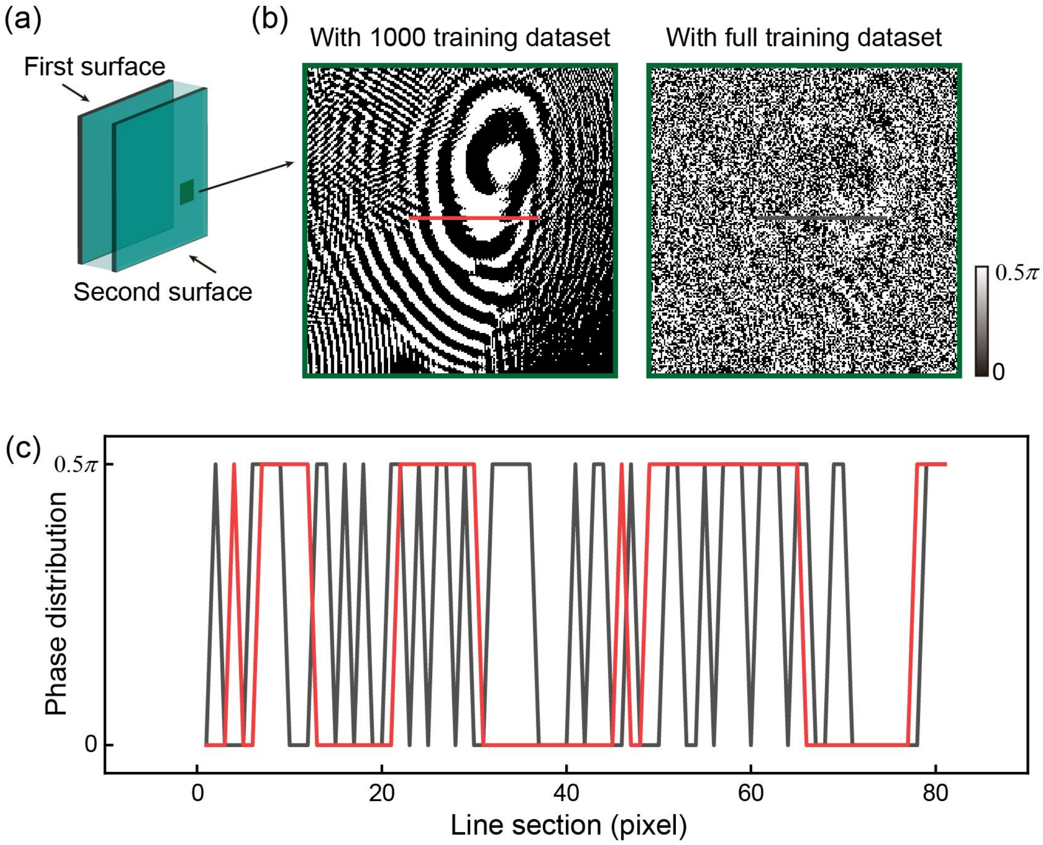


**Supplementary Figure 1. Frequency study of the phase distribution of two DNNs trained with datasets of 1000 images and 55000 images, respectively. a** Schematic illustration of the picked regions of the two DNNs. **b** Magnified phase distribution of the regions shown in a. Region size: $80 \times 80$ pixels. **c** Phase distribution along the horizontal lines shown in **b**.

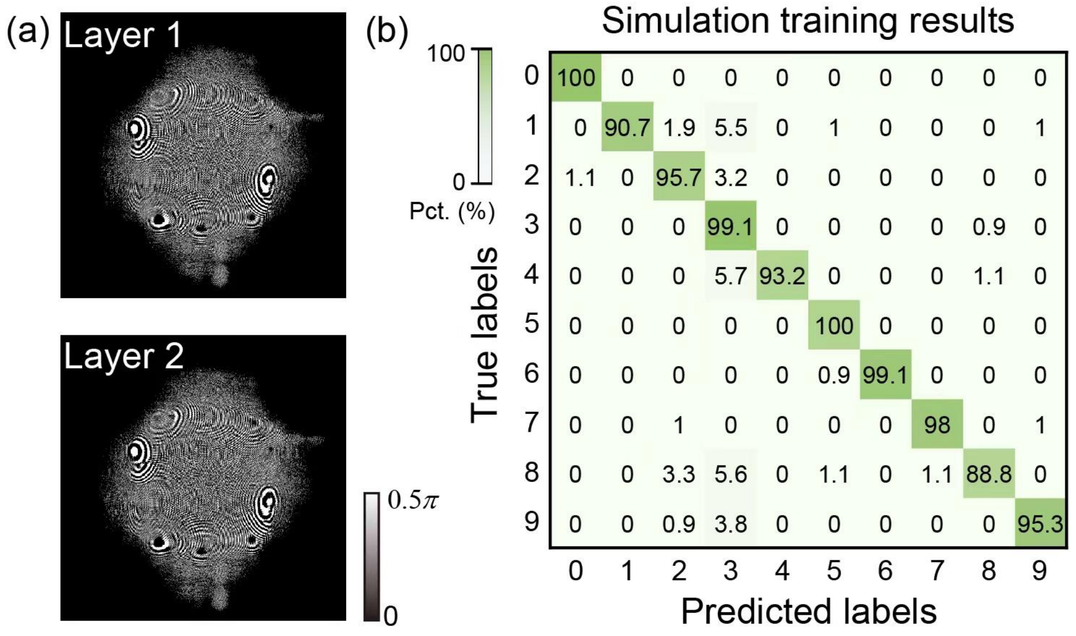


**Supplementary Figure 2. The designed bilayer DNN. a** Phase distribution of each layer. **b** Confusion matrix for the simulation results of the training set. 1000 different handwritten digits were used. Pct., percentage.

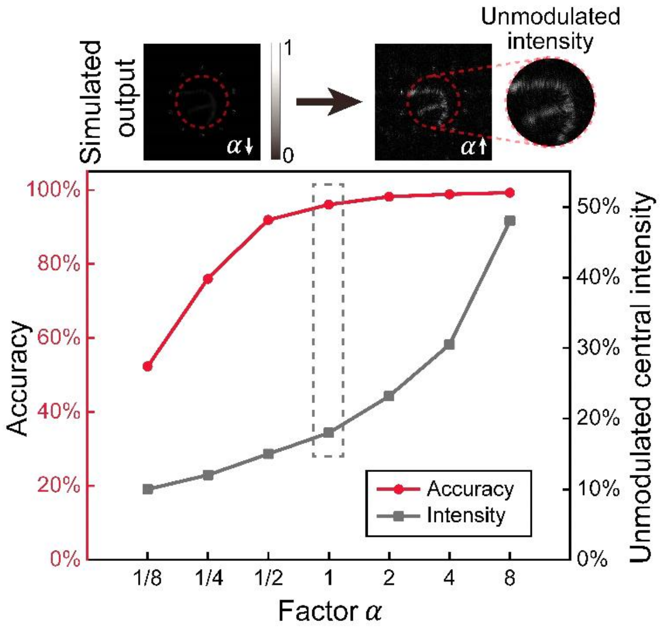


**Supplementary Figure 3. The accuracy and the unmodulated central image intensity versus the factor $\alpha$.**

Here, we added a factor $\alpha$ to the loss function, so the loss function can be written as follow.

$$\mathrm{MSE} = \frac{1}{n}\sum_{i=1}^{n} (\alpha I - I_i)^2$$

$I$ is the output of DNN and $I_i$ is the target intensity distribution at the output layer, so changing the value of $\alpha$ can modulate the light intensity of the 10 light spots corresponding to the 10 digits at the output layer. If the value of $\alpha$ decreases, the intensity of the ten light spots should be larger to have a small loss with the target intensity. Since the total input light intensity is constant, the increase of the intensity of the 10 light spots will lead to the decrease of the intensity of the digital image in the central area. Therefore, from Supplementary Figure 3, it can be seen that as $\alpha$ gradually decreases, the intensity of the digital image (unmodulated central intensity) will decrease, but the corresponding accuracy of DNN will also decrease. At the same time, the increase of $\alpha$ helps to improve the accuracy of DNN, but when the value of $\alpha$ is larger than 1, the improvement of the accuracy becomes very slow. Therefore, we chose $\alpha = 1$ in the training.

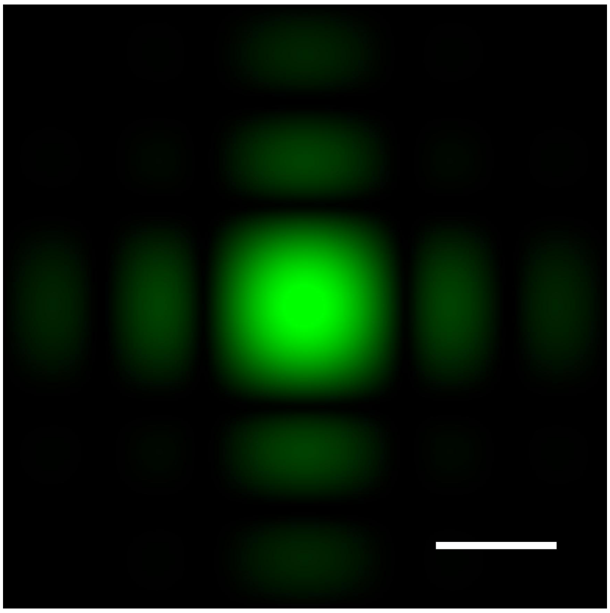

**Supplementary Figure 4**. **Diffractive pattern of one neuron in the 1$^{st}$ layer on the 2$^{nd}$ layer. Scale bar, 40 μm.**

We used an angular spectrum diffraction to simulate the diffraction of the light from one neuron in the 1$^{st}$ layer to the 2$^{nd}$ layer. Supplementary Figure 4 shows the result. We can see that the 0th order diffraction occupies most of the light energy, and the range of the 0th order diffraction is about 58×58 μm$^2$. Therefore, one neuron in the 1$^{st}$ layer is mainly connected with 7×7 neurons in the 2$^{nd}$ layer.

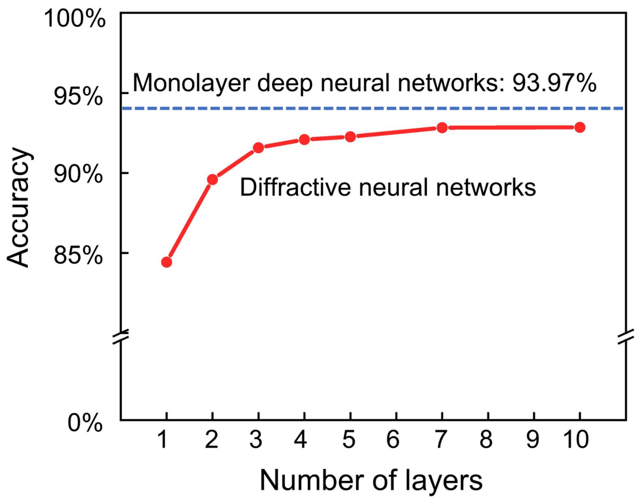


**Supplementary Figure 5**. **The training accuracy of DNN versus the numbers of layer.** The blue dotted line represents the accuracy of monolayer deep neural networks consistent with the parameters of DNN.

We studied the impact of numbers of layer on the accuracy of DNN. In the simulation, in order to reduce the amount of computation, we built a simpler DNN model. We set the number of neurons in each layer to be 28×28. The neuron size was set to 1 $\mu m^2$. The layer distance was set to 50 μm. The incident light wavelength was still set to 532 nm. 20000 handwritten digital images were used for training. We also simulated monolayer deep neural networks with the same number of elements (28×28). In order to compare with DNN, each weight element in the deep neural networks was set to be a complex value.

As can be seen from Supplementary Figure 5, as the number of layers increases, the accuracy of DNN gradually increases, but tends to a saturation value. The accuracy rate of 10-layer DNN is 92.85 %. Because the weight elements of deep neural network do not have the value constraints in the matrix column like DNN, the accuracy of monolayer deep neural networks can reach 93.97%. This result is consistent with the conclusion in Note 3.

This conclusion can help us estimate the accuracy limit of a DNN, that is, the accuracy of a monolayer deep neural network with the same number of neurons. It

should be noted that since the deep neural network does not have the physical parameters that used in DNN, like layer distance, neuron size and wavelength, this accuracy limit of DNN refers to the limit value after the number of layers, layer distance and neuron size of DNN are all optimized. This explains why the accuracy of the 10-layer DNN in Supplementary Figure 5 is still 1.12 % lower than that of the monolayer deep neural network. This is because the 50-μm layer distance and 1-$\mu m^2$ neuron size we randomly selected is not the best value, and there is still room for optimization.

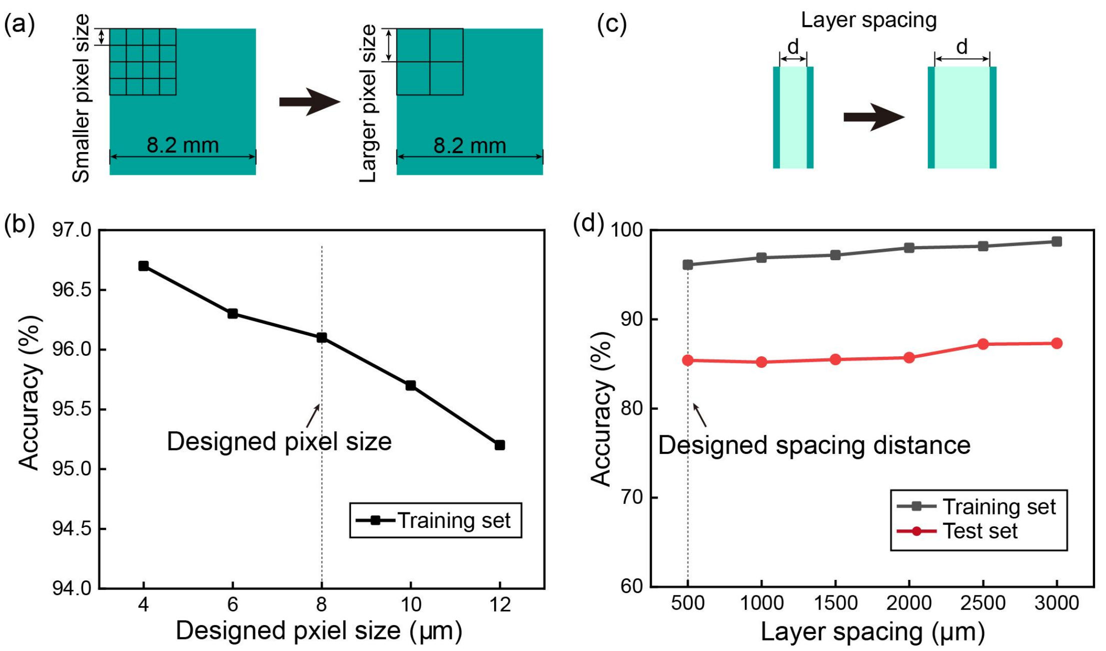


**Supplementary Figure 6. Simulation of the impact of neuron size change and layer spacing change on the performance of the DNNs chip.** A training set and a test set with 1000 images are used. **a** Illustration depicting DNNs with different pixel sizes while maintaining an identical chip size. **b** Comparison of training accuracy against varying pixel sizes. **c** Illustration of layer spacing change for DNN. **d** Accuracy changes under different layer spacing.

Supplementary Figures 6a and b demonstrate that accuracy improves as pixel size decreases. This is described by the equation, $sin\theta = m\frac{\lambda}{d}$, where, m donates the order of the diffraction, $\lambda$ represents the wavelength of the incident light, d refers to the spacing between the diffracting elements in the grating. A larger diffraction angle contributes to more connections between the two layers, thereby leading to higher training accuracy. Meanwhile, the layer spacing distance within the DNN chip, namely the gap between the double-side patterns, also plays a significant role. Supplementary Figures 6c and d illustrate that a larger layer spacing distance leads to better accuracy. This improvement is attributed to the wider gap improves the neuronal connection.

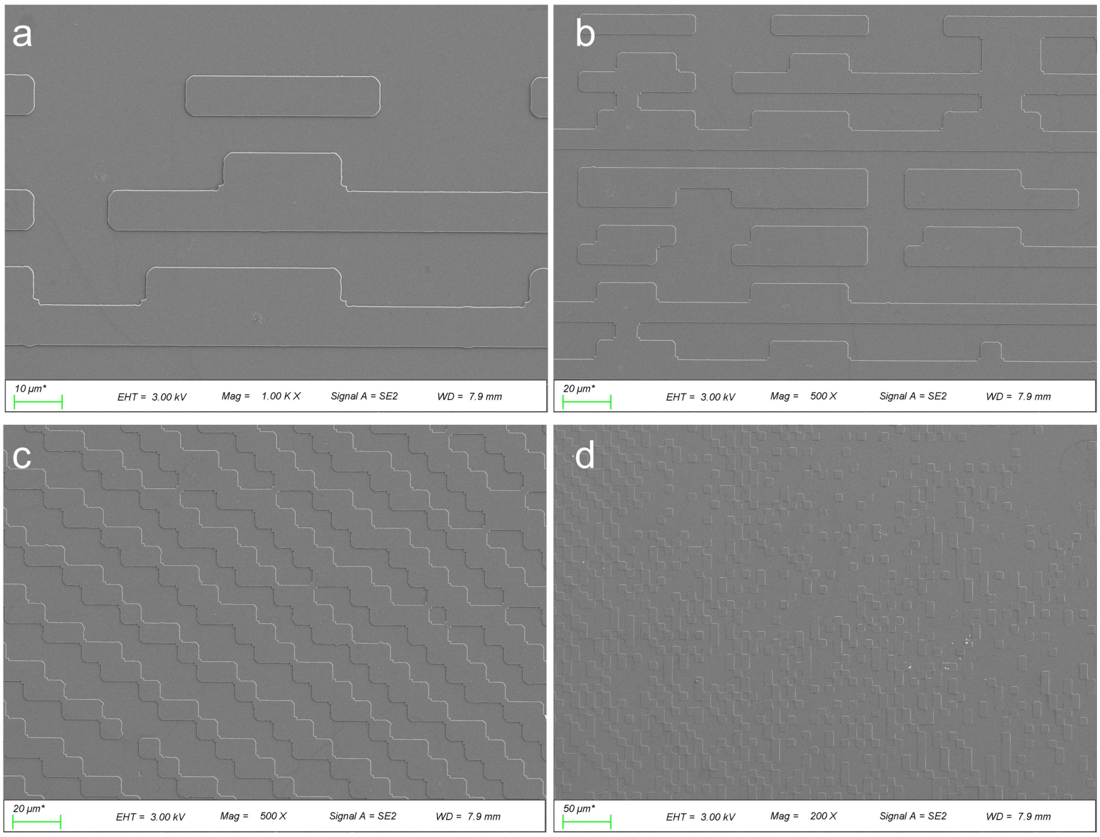


**Supplementary Figure 7. SEM images of the DNN with different magnification.**

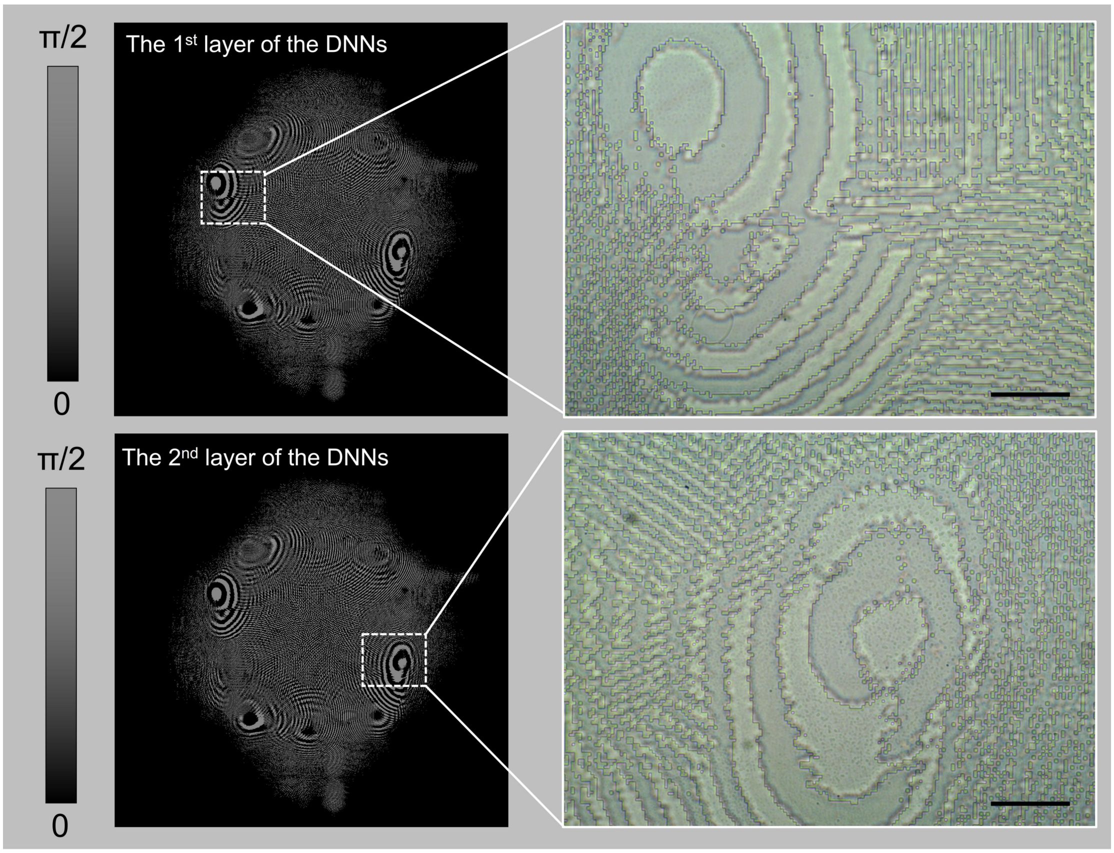


**Supplementary Figure 8. Optical images of the DNN. Scale bar, 200 μm. In the optical images, we can also see the blurry image presented by the pattern on the other side.**

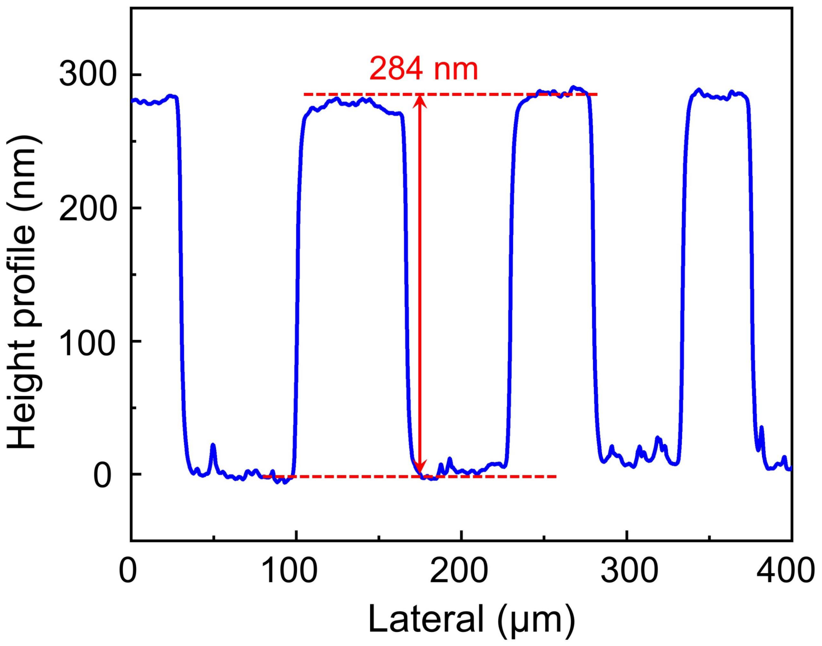


**Supplementary Figure 9. Height profile of the DNN.**

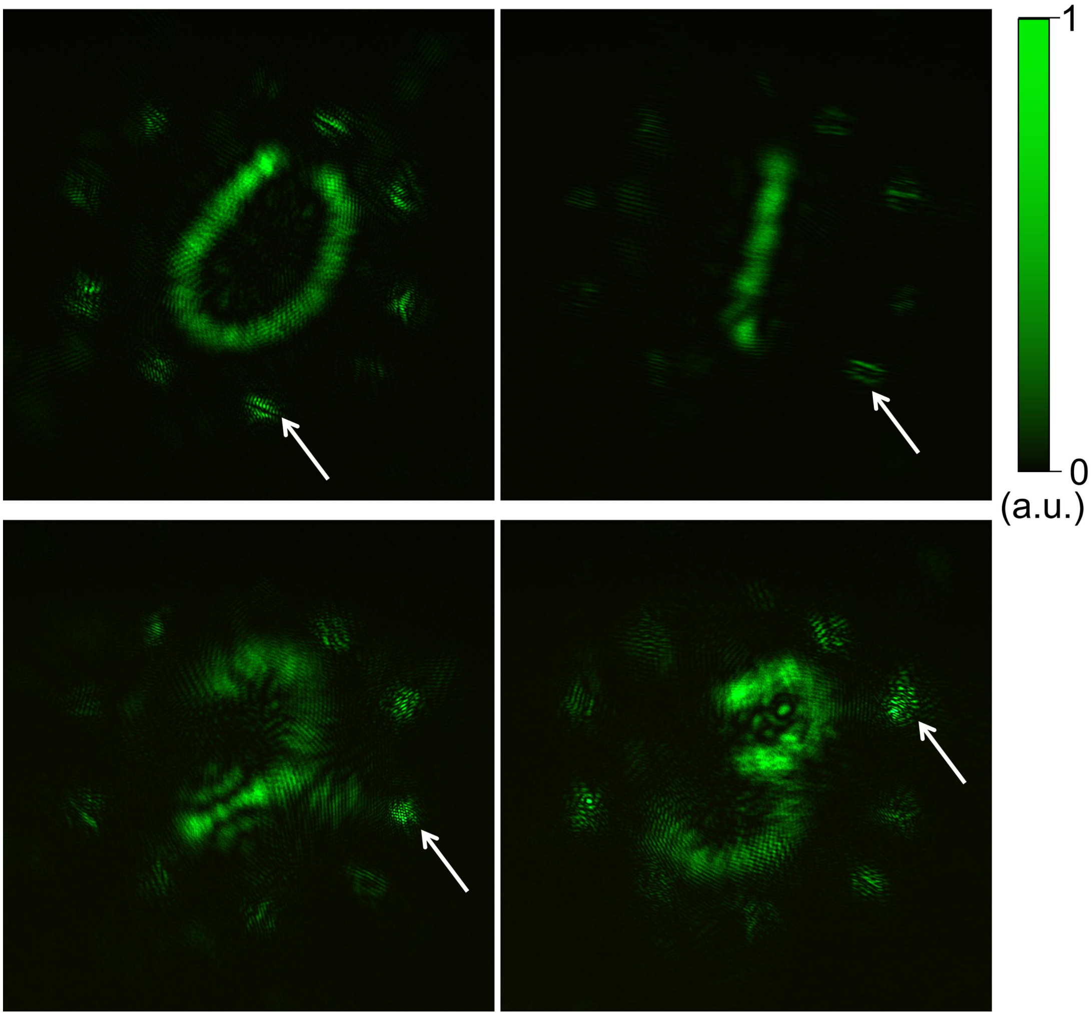


**Supplementary Figure 10. Enlarged optical images on the output layer. The white arrow points to the light spot with the largest light intensity.**

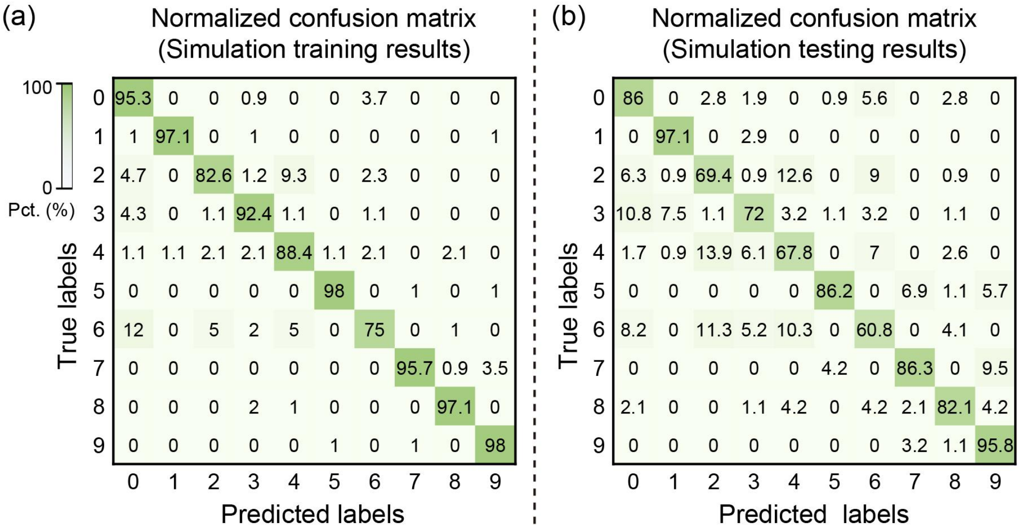


**Supplementary Figure 11. Simulated Fashion-MNIST recognition performance of the bilayer DNN chip.** A training set and a test set with 1000 images are used. a-b Normalized confusion matrixes of training set result and test set result, respectively.

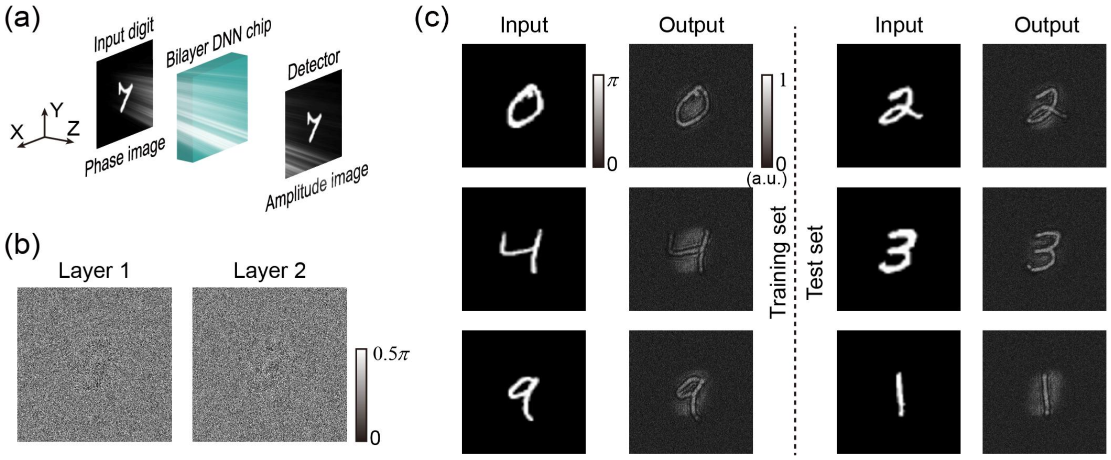


**Supplementary Figure 12. Simulated phase imaging performance of the bilayer DNN chip. a** Schematic of the simulation model. **b** Phase distribution of the DNN. **c** Training and test set simulation results for phase imaging. The inputs are phase-encoded handwritten digital images, and the outputs show light intensity distribution.

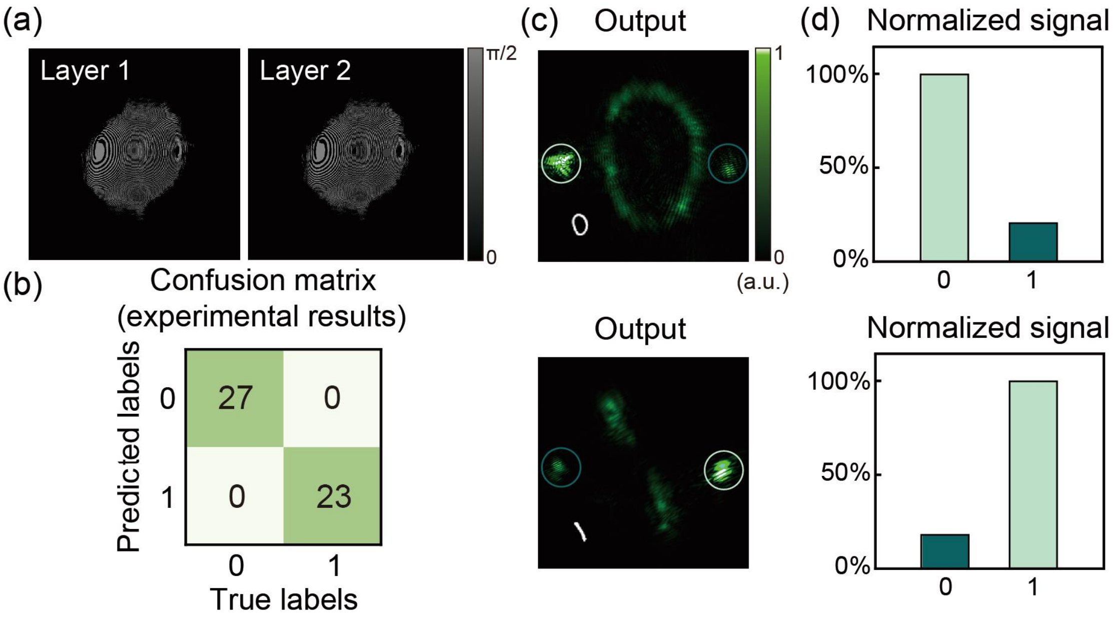


**Supplementary Figure 13. Experimental results of the bilayer DNN for recognition of handwritten digits 0 and 1. a** Phase distribution of each layer. **b** Confusion matrix for the experimental results of the test set (50 different handwritten digits). **c** Light intensity distribution on the output layer. **d** Normalized light intensity in the 2 circle areas shown in c.

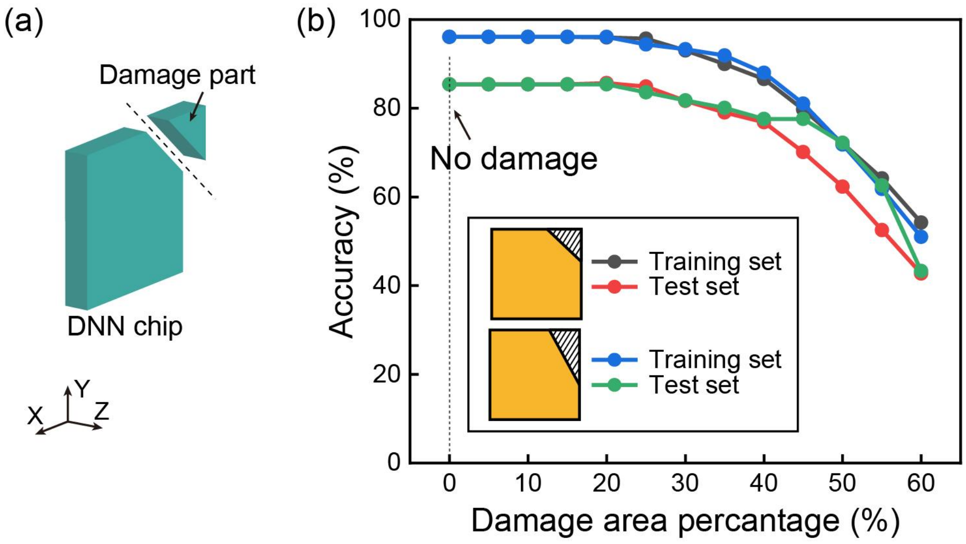


**Supplementary Figure 14. Simulation of the mechanical damage impact of DNN chip.** a Schematic of partial neuron loss in the sample caused by mechanical damage. b Accuracy of the DNNs changes with the damage area. The inset shows that we modeled two different shapes of damage.

For severe mechanical damage, a typical situation is that severe wear or sample fragmentation leads to the loss of part of neurons in the DNNs. We modeled this kind of mechanical damage by creating two different triangles in simulation (Supplementary Figure 14a). In Supplementary Figure 14b, the accuracy drop is depicted in relation to the increasing damaged area. Notably, the DNN chip can sustain its highest performance when the damage area remains below 20%. This resilience can be attributed to the relatively small size of the designed input digits, which is half the size of the chip. Consequently, damage to the corners of the DNN chip has a minimal impact on performance. However, as the damaged area expands, the accuracy experiences a more rapid decline. The descent in accuracy accelerates proportionally with the growth of the damaged region.

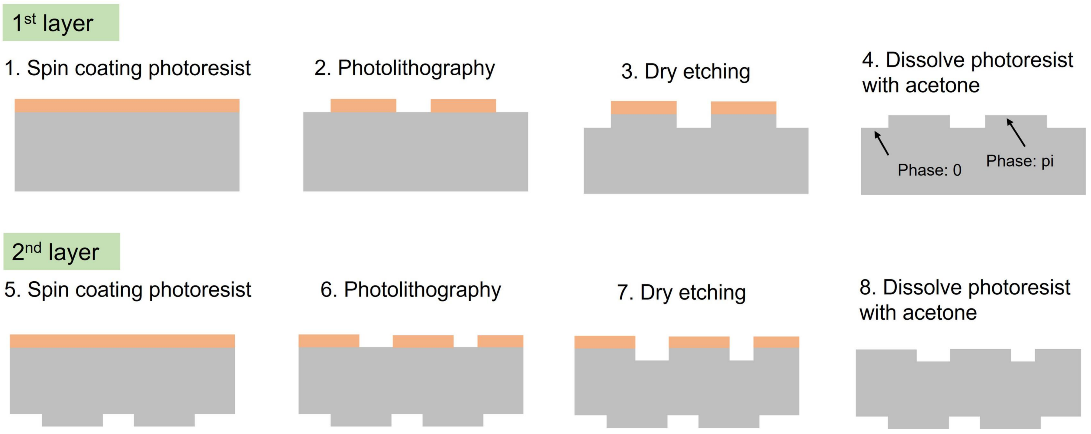


**Supplementary Figure 15. The fabrication process of the bilayer DNN.** It should be noted that when fabricate the second layer, it is necessary to align the pattern of the second layer with that of the first layer through the pre-designed registration mark.

**Supplementary Table 1. Performance comparison of different phase modulation levels.**

| | 256-level phase modulation | 2-level phase modulation |
|---|---|---|
| Accuracy | 96.3% | 96.1% |

We have simulated the DNN with 2-level phase modulation and 256-level phase modulation. From Supplementary Table 1, it can be seen that there is no significant difference in the accuracy of the two DNN.

**Supplementary Table 2. Performance comparison of the monolayer DNN and bilayer DNN.**

| | Monolayer DNN | Bilayer DNN |
|---|---|---|
| Loss function value | 0.4370 | 0.1983 |
| accuracy | 91.2% | 96.1% |

Supplementary Table 2 shows the simulation results of monolayer and bilayer DNN. Under the same other parameters, the accuracy of bilayer DNN is 4.9 % higher than that of monolayer DNN

**Supplementary Table 3. Comparison of the reported DNNs with this work.**

| Ref. | Implementation of DNN | Diffractive layers | Number of layers | Robustness of DNN | Tasks | Accuracy of tasks |
|---|---|---|---|---|---|---|
| 5 | Silicon meta-surface | Separated | 2 | Moderate | Digital recognition (6 types) | 90% |
| 6 | Nanofabrication on quartz plates | Separated | 5 | High | Digital recognition (10 types) | 84% |
| 7 | 3D laser printing in lithium niobate | / | 1 | Moderate | Odd and even classification (2 types) | 90% |
| 8 | $TiO_2$ meta-surface | / | 1 | Moderate | Digital recognition (4 types) | 93.75% |
| 9 | Si photonics | Integrated (2D integration) | 3 | Moderate | Iris plants classification (4 types) | 90% |
| 10 | 3D organic laser printing | Integrated (3D integration) | 4 | Low | Direct retrieval | / |
| This work | Double-sided nanofabrication on a quartz wafer | Integrated (3D integration) | 2 | High | Digital recognition (10 types) | 82% |

Note: Supplementary Table 3 shows the comparison of the reported DNNs with this work. Here we select some representative works on the fabrication or integration of DNNs. Their common features are that they all fabricated DNN samples in the experiment and the DNNs work in the visible or near-infrared band. When the work presented multiple tasks, we selected the one with similar tasks as our work. Compared to Ref. [6] with the same task, the accuracy of our DNN is lightly lower. This may be caused by the small number of training images and the less diffractive

layers. In terms of fabrication and integration of DNNs, our chip demonstrates high robustness and advances in 3D integration compared to other methods. The robustness analysis results are rough inferences based on the materials and fabrication methods of the DNNs.

**Supplementary References**:


1. Courbariaux M, Hubara I, Soudry D, El-Yaniv R, Bengio YJapa. Binarized neural networks: Training deep neural networks with weights and activations constrained to+ 1 or-1. arXiv:1602.02830 (2016).
2. Lin X*, et al.* All-optical machine learning using diffractive deep neural networks. *Science* **361**, 1004 (2018).
3. Binggeli N, Chelikowsky JR. Structural transformation of quartz at high pressures. *Nature* **353**, 344-346 (1991).
4. Wang B, Yu Y, Pignatelli I, Sant G, Bauchy M. Nature of radiation-induced defects in quartz. *The Journal of Chemical Physics* **143**, 024505 (2015).
5. He C*, et al.* Pluggable multitask diffractive neural networks based on cascaded metasurfaces. *Opto-Electronic Advances* **7**, 230005 (2024).
6. Chen H*, et al.* Diffractive Deep Neural Networks at Visible Wavelengths. *Engineering* **7**, 1483-1491 (2021).
7. Chen P*, et al.* Laser nanoprinting of 3D nonlinear holograms beyond 25000 pixels-per-inch for inter-wavelength-band information processing. *Nature Communications* **14**, 5523 (2023).
8. Luo X*, et al.* Metasurface-enabled on-chip multiplexed diffractive neural networks in the visible. *Light: Science & Applications* **11**, 158 (2022).
9. Fu T*, et al.* Photonic machine learning with on-chip diffractive optics. *Nature Communications* **14**, 70 (2023).
10. Goi E, Schoenhardt S, Gu M. Direct retrieval of Zernike-based pupil functions using integrated diffractive deep neural networks. *Nature Communications* **13**, 7531 (2022).
